# Photonic Crystal Structure and Coloration of Wing Scales of Butterflies Exhibiting Selective Wavelength Iridescence

**DOI:** 10.3390/ma5050754

**Published:** 2012-04-30

**Authors:** Filip Mika, Jiřina Matějková-Plšková, Suratwadee Jiwajinda, Punyavee Dechkrong, Makoto Shiojiri

**Affiliations:** 1 Institute of Scientific Instruments of the ASCR, v.v.i., Královopolská 147, Brno 612 64, Czech Republic; E-Mail: jirinanomat@gmail.com; 2 Bioresources and Biodiversity Section, Central Laboratory and Greenhouse Complex, Kasetsart University, Kamphaengsaen Campus, Nakhonpathom 73140, Thailand; E-Mails: rdiswj@ku.ac.th (S.J.); jibcidae134@gmail.com (P.D.); 3 Kyoto Institute of Technology, Kyoto 606-8585, Japan; E-Mail: shiojiri@pc4.so-net.ne.jp

**Keywords:** butterfly scale, structure color, natural photonic crystal, *E. mulciber*, *S. charonda*, *C. ataxus*, *T. aeacus*

## Abstract

The coloration of butterflies that exhibit human visible iridescence from violet to green has been elucidated. Highly tilted multilayers of cuticle on the ridges, which were found in the scales of male *S. charonda* and *E. mulciber* butterflies, produce a limited-view, selective wavelength iridescence (ultraviolet (UV)~green) as a result of multiple interference between the cuticle-air layers. The iridescence from *C**.** ataxus* originates from multilayers in the groove plates between the ridges and ribs. The interference takes place between the top and bottom surfaces of each layer and incoherently between different layers. Consequently, the male with the layers that are ~270 nm thick reflects light of UV~560 nm (green) and the female with the layers that are ~191 nm thick reflects light of UV~400 nm (violet). *T. aeacus* does not produce the iridescent sheen which *T. magellanus* does. No iridescent sheen is ascribed to microrib layers, which are perpendicular to the scale plane, so that they cannot reflect any backscattering. The structures of these butterflies would provide us helpful hints to manipulate light in photoelectric devices, such as blue or UV LEDs.

## 1. Introduction

Characteristic patterns and the vivid coloration of the wing scales of butterflies have lately attracted considerable attention as natural photonic crystals. Earlier work on the structural colors of butterfly wings can be seen in a series of optical microscopy [1,2,3] and electron microscopy [4]. A vivid coloration of butterfly wing scales would be based on pigments or microstructures (chemical and physical colors) or both combined [5,6]. The melanin and pterin pigments frequently found in butterflies can produce yellow, red, black and brown colors, while pure pigments cannot produce blue, violet, green and golden colors [6].

An Indonesian male *Papilio palinurus* butterfly displays bright green hues due to the extraordinary combination of both yellow and blue iridescence, which arises from modulated multilayers with a blue component as the result of an orthogonal-surface retro-reflection process [5,7]. A Costa Rican male *Ancyluris meliboeus* (*A. meliboeus*) butterfly, called a ‘living jewel’, produces bright iridescence of a broad wavelength range by the highly tilted, multilayered arrangement in the ventral wing scales, and generates a strong flicker contrast with minimal wing movement [8]. A *Troides magellanus* (*T. magellanus*) butterfly, called ‘Magellan birdwing’ and inhabiting the Philippines and Taiwan, exhibits a blue-green sheen on the hindwings when both illuminated and viewed at near-grazing incidence. Lawrence *et al.* [9] reported in 2002 that the *T. magellanus* uses pigment coloration at all but a narrow tailored range of angles, where multilayered rib-like (or microrib) scales cause the characteristic effect, adding a blue-green sheen that had been known in only one other species of butterfly. It was the *A. meliboeus* [8,9]. This unique visual attraction of the *T. magellanus* was detailed taking into account correlated diffraction and fluorescence in the backscattering iridescence [10]. Recently, we have observed *Troides aeacus* (*T. aeacus*) that belongs to the same genus as *T.* *magellanus* [11]. The *T. aeacus* has microrib layers normal to the wing plane. The microrib layers cannot cause the backscattering diffraction for any incident light, and consequently does not exhibit iridescence, which *T. magellanus* produces with tilted microrib layers. We have also found a highly tilted, multilayered arrangement in vivid iridescent scales in a male *Sasakia charonda* (*S. charonda*) butterfly [12,13]. The structure of its scales is similar to that in the *A. meliboeus*. The iridescence is caused by the interference of incident light reflected with a kind of blazed diffraction grating of the scales that has a high efficiency in a shorter wavelength range of 200~450 nm. We have investigated the coloration in different areas in the wings of a male *Euploea mulciber* (*E. mulciber*) butterfly [11], and male and female *Chrysozephyrus ataxus* (*C. ataxus*) butterflies [14]. They are famous for vivid violet-green iridescent wings, similar to *A. meliboeus* and *S. charonda*.

Lepidoptera, which is the generic name for butterflies and moths, has about 180,000 species in the world. Different structural colors of the wings have been studied for various butterflies and moths, e.g., Lycaenide butterflies [6,15,16], twelve species from four families [17], *Colias eurytheme* L [18], *Morpho cypris* [19], *Chrysiridia rhipheus* (Madagascan sunset moth) [20,21], *Pontia protodice*, *Colias eurytheme* [22], *Pieris rapae* (small white) [23], and others [24,25]. The structures of the photonic crystals of the wing scales would be applicable to fine light manipulators such as reflection elements in light-emitting devices. In fact, the photonic structures of butterfly scales have been prototyped using atomic layer deposition [26,27] and a biotemplate method [28]. Therefore, the structural investigations of the butterfly scales are still required for achieving tunable photonic properties in artificial scales.

The present paper reviews recent investigations of the microstructure and coloration of the scales of butterflies that exhibit selective iridescence from violet to green. It is dwelt on why human eyes observe only the limited iridescent hues from such butterflies as *E.* *mulciber*, *S. charonda* and *C. ataxus*.

## 2. Samples and Methods

**Figure 1:**
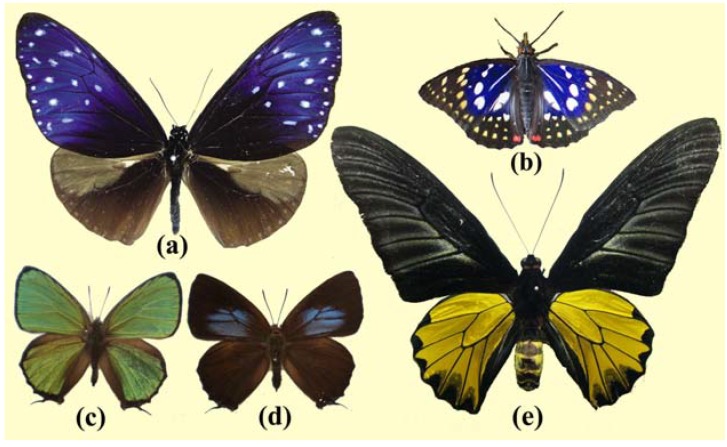
(**a**) Male *E. mulciber*; (**b**) Male *S*. *charonda*; (**c**) Male *C. ataxus*; (**d**) Female *C. ataxus*; (**e**) Male *T. aeacus*.

The *E. mulciber* butterfly, called the ‘striped blue crow’, is a common butterfly in Thailand, Malaysia, Singapore, Laos, Vietnam, South China, *etc*. (Figure 1(a)). It is in the subfamily Danainae of the family Nymphalidae. Its wingspan is 80–90 mm. The dorsal side of the male’s forewing is dark brown strongly adorned with iridescent blue and numerous white spots. The hindwing is dark brown with a prominent pale brown coloring at the costal half and a small grey patch [29]. The male *E. mulciber* (and the male *T. aeacus*) butterflies used in this experiment were reared from eggs at the Environmental Entomology Research & Development Center, Kasetsart University.

The *S*. *charonda* is a species of butterflies in the Apaturinae subfamily of Nymphalidae, and is called the ‘great purple emperor’ in English and ‘Ohmurasaki’ in Japanese (Figure 1(b)). It is found in the woodlands in Japan as well as in China, Korea, Taiwan, and Vietnam. The wingspans of the male and female are ~50 and ~65 mm, respectively. Adults have dark brown wings with white patches and a small orange spot on each of the hindwings. The male generates purple-blue iridescence in the forewings and hindwings, while the female lacks this characteristic. We sampled them in Nagano Prefecture in the middle of Japan.

*C. ataxus* belongs to the subfamily of Theclinae in the family of Lycaenidae. The *C. ataxus* has another name—*Thermozephyrus ataxus—*because *Chrysozephyrus* is a synonym of *Thermozephyrus*. It inhabits Japan as well as the southwest of China and the northwest district of the Himalayas. Its wingspan is around 38~42 mm. The dorsal surfaces of the male wings are metallically glittering green-violet with very narrow sharp black borders (Figure 1(c)), and their ventral surfaces are silver white with several pale brown specks and a few orange-ringed spots at the hindwing ends. The dorsal surfaces of the female’s forewings are dark brown with violet marks (Figure 1(d)), and their ventral surfaces are brown with white bands. In the present experiment we used a male and a female *C. ataxus* butterfly of a subspecies of *Kirishimaensis* (called ‘Kirishima-midori-shijimi’), which had been reared from eggs sampled at Kaminyu, Shiga, Japan.

*T. aeacus*, called the ‘golden birdwing’, belongs to the subfamily Papilioninae of the family Papilionidae. It is also a common butterfly found in Thailand, Nepal, India, Myanmar, Laos, Cambodia, Vietnam, and west China. It has a wingspan reaching about 15–16 cm. Its hindwing is yellow with black markings, including marginal spots along the margins and having black dusting on the inner edges of marginal spots, especially in males, as seen in Figure 1(e).

Scanning electron microscopy (SEM) observations were performed in conventional secondary electron detection mode using field-emission scanning electron microscopes. The wings were coated with a sputtered gold layer about 10–20 nm thick to avoid charging effects. Some scales were removed from the wings without any coating and observed in their authentic intact natural state. The hues of the scales’ colors were examined in an optical microscope (OM). Reflectance of the wing scales was measured by using an opto-spectrometer (Perkin Elmer Lambda 900) with two light sources of variable wavelength ranges of 200~375 nm and 375~2,500 nm. Two detectors for 200~860.8 nm and 860.8~2,500 nm were used. To study the localized optical property of wing, the incident beam along the wing normal was focused to 2 mm^2^.

## 3. Results and Discussion

### 3.1. Highly Tilted Multilayers of Cuticle on the Ridges

**Figure 2:**
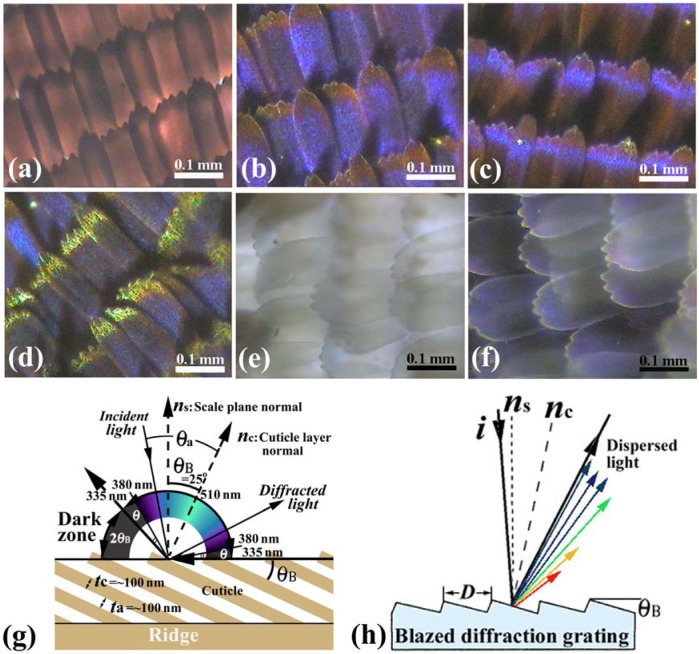
(**a**–**f**) Optical microscope (OM) images of the scales of the male *E. mulciber*, taken at different incident angles of white light. Transmission image (**a**) and reflection images of **B** scales (**b**–**d**); Transmission image (**e**) and reflection image of **W** scales (**f**); (**g**) Schematic illustration of the selective reflection from cuticle layers piled on the ridge; (**h**) Schematic illustration of a blazed diffraction grating (adapted from [11]).

Figure 2(a–d) shows OM images of scales in a vivid blue iridescent area in the forewing of the male *E. mulciber*. As seen in a transmission image taken with white light (Figure 2(a)), these scales have the intrinsic dark brown color due to their melanin-content. When the images of the scales are taken under reflection mode, they exhibit a vivid blue-green iridescence only in parts where the incident light is reflected on the surfaces, as seen in Figure 2(b–d).

**Figure 3:**
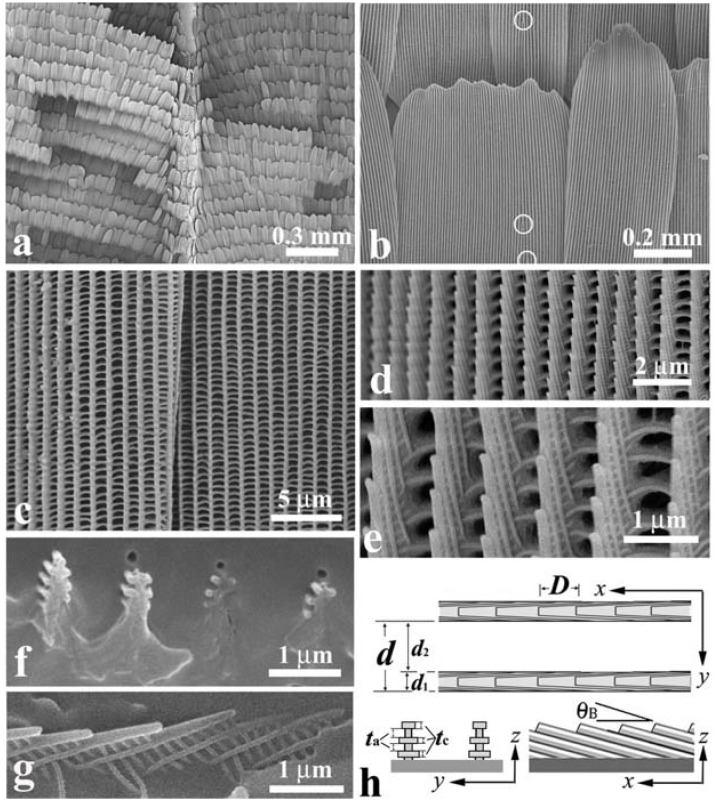
Scanning electron microscope (SEM) images of scales in the forewing of the male *E. mulciber*. (**a**) Scales on a vein and **B** scales in the blue-iridescent background; (**b**) **B** scales; (**c**) Top view of two **B** scales; (**d**,**e**) Enlarged images of ridges in the **B** scale; (**f**) Cross-section of ridges on a y-z plane; (**g**) Cross-section on an x–z plane; (**h**) Schematic of the piled cuticles (adapted from [11]).

Figure 3(a) shows an SEM image of scales on a vein and the surrounding blue iridescent background. The iridescent blue background comprises two kinds of scales: Broad and narrow scales, which are almost alternately arranged so that the spaces between the broad scales are covered with the narrow scales, as can be clearly seen in Figure 3(b). Figure 3(c) shows a top view of the ridges in a narrow scale (left) and a broad scale (right). The ridges run along the length of the scale. The ridge occasionally branches, as indicated by the circles in Figure 3(b), looking like the edge dislocation in a crystal. This is a kind of growth defect of the living tissue, as previously reported in *S. charonda* [12]. The scales on the vein are almost the same in shape with the narrow scales in the background. Figure 3(d–g) reveal a multilayered arrangement of cuticles on the ridges, which is similar to that discovered in *A. meliboeus* [8] and that observed in *Morpho Peleides* [26] and *S. charonda* [12]. The scales form a three-dimensional optical diffraction grating. Figure 3(h) illustrates schematic projections of the grating, which is composed of the grid of the ridges with the spacing *d*, the *n* multilayered arrangement of cuticles lapped on the ridge, and the surface arrangement of cuticles tilted at *θ*_B_ and spaced by *D*. The *x*-axis is defined along the ridges running the length of the scale from the root, the *z*-axis is normal to the scale plane and the *y*-axis normal to the *x*–*z* plane. The width of the ridges *d*_1_ and the width of the grooves *d*_2_ as well as the spacing *d* and *D* are estimated and listed in Table 1. We also estimated the thickness of the cuticle layers and air gaps to be *t*_c_ = ~100 nm and *t*_a_ = ~100 nm, respectively, and the tilting angle of *θ*_B_ to be ~25°. The number of the piled cuticle layers is *n* = 3, which is smaller than 7 in the *S. charonda* [12] and 4 in the *A. meliboeus* [8].

The broad and narrow scales are almost the same in microstructure, and we will call them **B** scales hereafter. The scales in the white spots (**W** scales) resemble the broad **B** scales in shape and structure but not in color. They are transparent white or pearl due to little content of melanin pigment, as seen in Figure 2(e) which is a transmission OM image. The **W** scales also exhibit the iridescent hues when they reflect the incident light, as seen in Figure 2(f). The parameters of the grating of the **W** scales were measured and are shown in Table 1.

**Figure 4:**
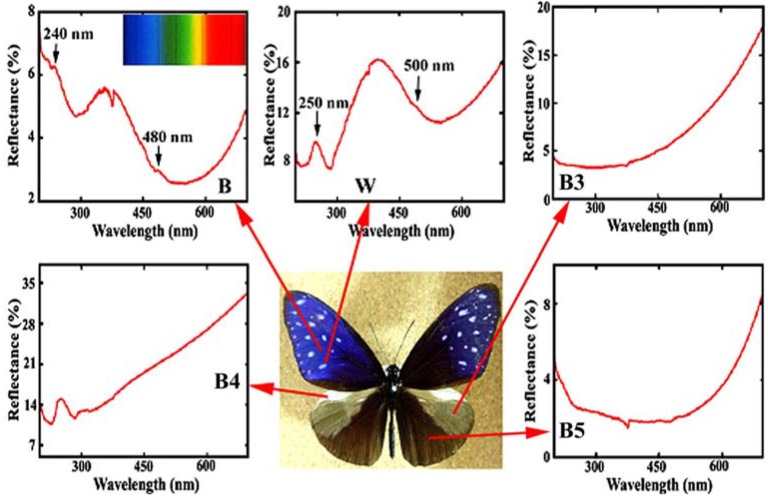
Reflectance spectrum (UV and visible region) from different areas in the dorsal wings of the male *E.* *mulciber.* Small caves at 375 nm were caused by a change of the incident light source so that they should be neglected. Inset is the spectrum of sunlight (adapted from [11]).

Vukusic *et al*. [8] illustrated that the layer tilt of *θ*_B_ = 30° causes a 60° portion of the wing’s ‘observation hemisphere’ not to appear leading to an iridescent ‘dark zone’ in *A. meliboeus*. The dark zone (2*θ*_B_ = 50°) corresponds to the *E.* *mulciber*’s scales and is schematically shown in the Figure 2(g). The dark area in the right wing of the butterfly shown in Figure 4 is surely in the dark zone. The butterfly can thereby generate a strong flicker contrast with minimal wing movement. They also addressed that the diffraction component appears to combine additively with the interference from the underlying multilayer to produce a broad range of coloration, as well as a limited reverse color change with an angle compared to that associated with conventional flat multilayering [8]. It may be considered that the grid, which has the spacing of *d* along the *y* axis and the spacing of *D* along the *x* axis, and the tilted triple cuticle/air layers with the spacing of *t*_c_ *+* *t**_a_*** form a three-dimensional grating or a monoclinic lattice.

**Table 1:** Diffraction grating of the wing scales in butterflies.

Buterfly	Scale	*d*_1_ (μm)	*d*_2_ (μm)	*d* (μm)	*D* (μm)	*d*_1_/*d* (%)	*n*	*t* (μm)	*θ*_B_ (°)	Reference
*E. mulciber*	**B**	0.3~0.4	0.6~0.7	0.9~1.1	~0.5	~35	3	*t*_c_ = ~0.1	25	
	**W**	~0.25	0.8~0.9	1.0~1.2	0.5~0.8	~23		*t*_a_ = ~0.1		[11]
	**B4**	~0.2	~1.3	~1.5	0.6~ 1.7	~13	1			
*S. charonda*	**B& W**	~0.6	~0.3	~0.9	~1.0	~67	7	~1	~8	[13]
	**B1, W1 & R1**	~0.4	~1.1	~1.5	~0.8	~27	2	~0.8	~10	
*C. ataxus* M	Green-blue	0.4~0.5	2.5	3	0.8~1.5	~15	1	*D’* = 0.8~1.2		[14]
F	Violet	~0.25	~2.1	~2.3	~1.4	~11	1	*D’* = ~0.9		
	Dark brown	~0.3	~1.2	~1.5	~2.2	~20	1	*D’* = ~0.6		

M and F are male and female. *d*_1_, *d*_2_, *d*, *D* and *t* (or *t*_c_ and *t*_a_*)* are indicated in Figure 3(h). *n* is the number of cuticle layers piled on the ridge. *θ*_B_ is the angle between the cuticle and scale surface. *D’* is the spacing between the cross ribs.

Figure 4 reproduces the reflectance in the ultraviolet (UV) and visible region from the different areas in the dorsal wings of the male *E.* *mulciber.* The spectrum from the dark brown scales **B** exhibiting iridescent blue has a heap with high reflectance of 4~6% in a range over UV (<380 nm) and violet (380~450 nm), and a valley with lower reflectance below 4% in a range over blue (450~495 nm), green (495~570 nm), yellow (570~590 nm), and orange (590~620 nm). Small peaks can be seen at ~480 and ~240 nm. The vivid blue coloration comes from the multiple cuticle layers on the ridges. For simplicity we assume the incident and reflected rays in the *x*-*z* plane. From the cuticle-air multilayered arrangement the multiple reflection occurs as dynamical diffraction or coherent reflection between layers, when the following interference condition is satisfied:

2(*n*_a_*t*_a_ cos*θ*_a_*+n*_c_*t*_c_ cos*θ*_c_) = *mλ*_p_ (1)

where *θ*_a_ and *θ*_c_ are the angles of incidence and refraction of the rays to the cuticle layer normal, *n*_a_ and *n*_c_ are the relative refractive index of air and cuticles, respectively. An integer of *m* is the order of interference and *λ*_P_ is the wavelength of the reflected light. Since in the reflectance measurement the incident rays were parallel to the scale plane normal (which is different from the cuticle layer normal as illustrated in Figure 3(g)), *θ*_a_ = *θ*_B_ and then sin*θ*_B_/sin*θ*_c_ = *n*_c_. Taking *n*_c_ = 1.55 as an appropriate value for the cuticles [20,21], *n*_a_ = 1, *t*_c_ = ~100 nm, *t*_a_ = ~100 nm, and *θ*_B_ = 25°, we can obtain *λ*_p_ = ~480 nm for *m* = 1 and *λ*_p_ = ~240 nm for *m* = 2. These wavelengths correspond to the observed peaks in the spectrum. As a result, this confirms the assumption of *n*_c_ = 1.55.

According to the dark zone mentioned above, the incident angle *θ*_a_ which causes the observed reflection, is limited within 90° − *θ*_B_ = 65° > *θ*_a_ > −65° = −90° + *θ*_B_. Since sin*θ***_a_**/sin*θ*_c_ = *n*_c_ = 1.55, the wavelength of the reflected rays *λ* must be ~510 > *λ* > ~335 nm for *m* = 1 and ~255 > *λ* > ~168 nm for *m* = 2, because the wavelength calculated from Equation (1) is *λ*=335 nm at *θ*_a_ = ±65° and *λ* = 510 nm at *θ*_a_ = 0 for *m* = 1, and *λ* = 168 nm at *θ*_a_ = ±65° and *λ* *=* 255 nm at *θ*_a_ = 0 for *m* = 2. Hence, human eyes, which respond to wavelengths from about 380 to 790 nm, can see the iridescent reflected rays only in a range from ~510 nm (green) to ~380 nm (violet), which is schematically illustrated in Figure 2(g). The violet and green hues are observed in reflections from the scales in Figure 2(b–d) and also Figure 2(f). The incident (and reflection) angle *θ*_a_ corresponding to *λ* = 380 nm for *m* = 1 can be calculated to be ±54.5°. Therefore, besides the dark zone where no reflection geometrically occurs [8], human invisible zones due to UV reflection appear in an angle of *θ* = 10.5° (= 90° − 25° − 54.5°) at both sides of the visible zone, as shown in Figure 2(g). If the incident ray is not in the *x*–*z* plane, we can explain the reflection using the Ewald construction for the monoclinic lattice mentioned above. A diffractogram of a **W** scale removed from the white patch in the male *S. charonda* wing, may be regarded as a cross-section of the reciprocal lattice space used in the Ewald construction.

The tilting of the cuticles on the ridges, indicated in Figure 3(h), forms a blazed grating. The blazed diffraction grating, illustrated in Figure 2(h), is designed to obtain high diffraction efficiency for a certain order *m* and wavelength [30]. When the incident light and the *m*-th order diffracted light are related by mirror reflection with each other on the facet surfaces, most of the incident energy is concentrated into the *m*-th order diffracted light. This satisfies λ = (2*D*/*m*) sin*θ*_B_ cos (α−*θ*_B_), where *D* is the spacing or the grating period and α is the angle between the incident light ***i*** and the grating normal ***n***_s_. The angle *θ*_B_ is called blaze angle. The wavelength for *m* = 1 and α = *θ*_B_, where the 1st-order diffracted light returns along the same path as the incident light, is called the blaze wavelength *λ*_B_, and then *λ*_Β_ = 2Dsin*θ*_B_. The blaze wavelength represents the blaze characteristics of the grating. For the **B** scales *D* = ~0.5 μm and *θ*_B_ = ~25° so that the blaze wavelength is estimated to be *λ*_Β_ = ~400 nm. The high diffraction efficiency from this multilayered grating hence would be obtained in a low wavelength range of violet, which may be a reason of the heap of the spectrum in Figure 4(a). Thus, the reason why the *E.* *mulciber* butterfly wings exhibit vivid iridescent violet hues has been completely elucidated.

The reflectance of a white spot with **W** scales, shown in Figure 4, is greater than that of **B**, and exceeds 10%, corresponding to its white hues. Heaps appear with peaks at ~500 and ~250 nm. **B** and **W** scales have almost the same microstructure and exhibit similar peaks in the reflectance spectra. Therefore it may be considered that the vivid blue coloration of **B** is caused by melanin in the cuticle layers. According to Ou-Yang *et al.* [31], the typical absorbance spectrum of soluble eumelanin includes a linear increase of absorbance from 800 to 600 nm and an exponential increase of absorbance from 600 to 300 nm. In any case the strong reflection is caused from areas satisfying the interference condition, where the semitransparent brown cuticle layers also work as internal optical feedback reflectors. The parts that are out of the interference condition look dark brown due to the absorption of shorter wavelength rays in the incident rays (see Figure 2(b–d)), reducing scattering light and working only as a dark background to make the reflection light stand out.

The reflectance spectra of other areas are also shown in Figure 4. The scales in these areas were observed by SEM. The grey patch marked by **B3** has long flat fiber scales like sea tangles. The scales in the prominent pale brown costal area **B4** are dark brown with deep splits on the end and exhibit no iridescence with monolayer cuticle arrangement (see Table 1). The scales in the **B5** area are dark brown, similar to **B** and **W** scales but not the same because of a double-layers arrangement of cuticles on the ridges. The spectrum of **B5** indicates that the double-layer arrangement is not enough to reflect observable interference light. The details have been reported in [11].

**Figure 5:**
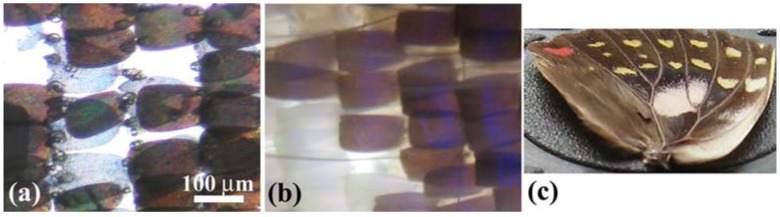
(**a**,**b**) OM images of scales on the border between the purple-blue iridescent area and white patch in a forewing of the male *S. charonda*. Transmission image (**a**) and reflection image (**b**); (**c**) The hindwing taken at a low glancing angle under sunlight (adapted from [13]).

Next, we brief the investigation on the *S. charonda* butterfly, comparing it with and complimenting the information found on *E. mulciber* mentioned above. Figure 5(a) shows a transmission OM image of scales on the border between the purple-blue iridescent area (**B**) and white patch (**W**) in a forewing of the male butterfly. Figure 5(b) is a reflection image. The observed brown scales **B** and transparent scales **W** seem very similar in optical property to the **B** and **W** scales of the male *E. mulciber*, respectively.

**Figure 6:**
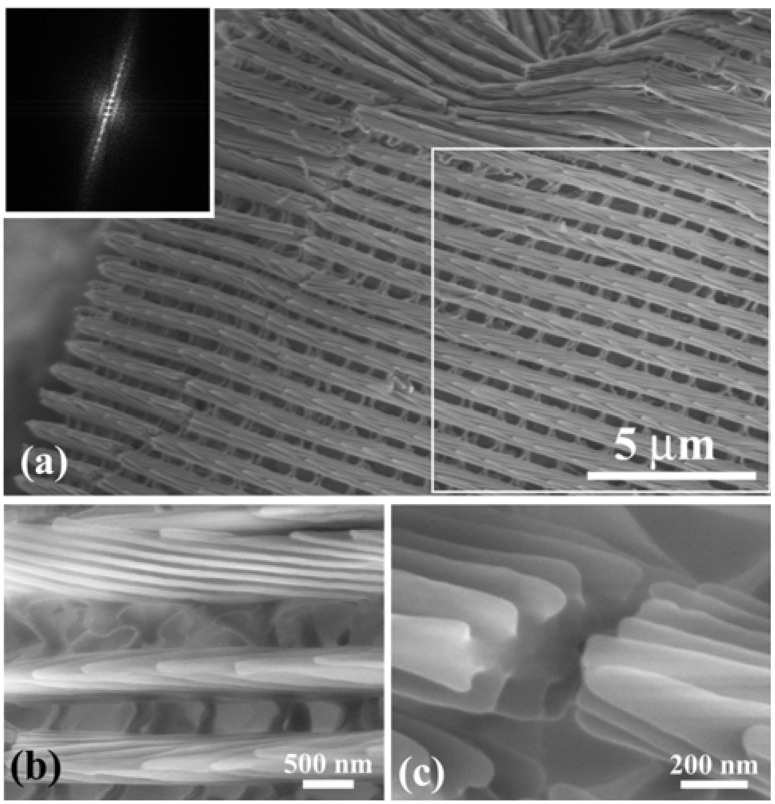
(**a**) SEM image of a **W** scale removed from the white patch in the male *S. charonda* wing. The inset is a computer diffractogram from the area enclosed by the square; (**b**) The side view and top view of the cuticles piled on the ridges; (**c**) The cross-sectional view of cuticle layers. **B** scales in the purple-blue iridescent area have the same microstructure (adapted from [13]).

Figure 6 shows SEM images of the **W** scales that were removed from the wings. During the preparation they were bent and broken, and thus allowed us to observe the side and broken cross-section as well as the top of the cuticles lapped on the ridges. The images reveal the highly tilted, multilayered arrangement and the three-dimensional grating structure of the **W** scale. The SEM observation also showed that the **B** and **W** scales are the same in microstructure. The difference between the **B** and **W** scales is only the content of melanin. The parameters of the diffraction grating of these scales are shown in Table 1 [13]. The **B** and **W** scales of the male *S. charonda* have seven cuticle layers piled on the ridges, that is *n* = 7. They also have the wide ridges, which is recognized from a ratio *d*_1_/*d* of ~2/3 as compared with ~1/3 for the **B** scales of *E. mulciber* (see Table 1). The seven cuticle layers on the wide ridges would cause a stronger iridescence—due to multiple interference—than found with *E* *mulciber* (*n* = 3) and *A. meliboeus* (*n* = 4). For the scales of *S. charonda*, the tilting angle of cuticle layers or the blaze angle is *θ*_B_ = 8° so that the dark zone shown in Figure 3(g) is within 2*θ*_B_~16°. A photograph of the hindwing taken at a low glancing angle under sunlight is shown in Figure 5(c), which clearly indicates the dark zone.

The **B1**, **W1**, and **R1** scales, which are named for the scales in the dark brown area, the yellowish patches and the small orange spots, respectively, have the same microstructure (Table 1). The difference is ascribed to the species and quantity of the contained melanin; perhaps brown-black dihydroxyindole eumelanin and red-brown benzothiazine pheomelanin. The small ratio of *d*_1_/*d = ~*0.27 and the number of the piled cuticle layers of *n* = 2 may be insufficient to develop the iridescence hues. Rather, the incident light, particularly UV radiation, may be absorbed and converted to heat energy by melanin. The female butterflies have bigger brown wings without iridescent scales, which significantly reduce the penetration of light due to reflection. The females thereby can receive more heat energy, to be used for breeding, from sunshine than the male butterflies. Thus, the wing structure of the butterflies may give us a hint to design photochemical crystal devices for internal conversion or radiationless de-excitation where the UV radiation is transformed into heat [13].

**Figure 7:**
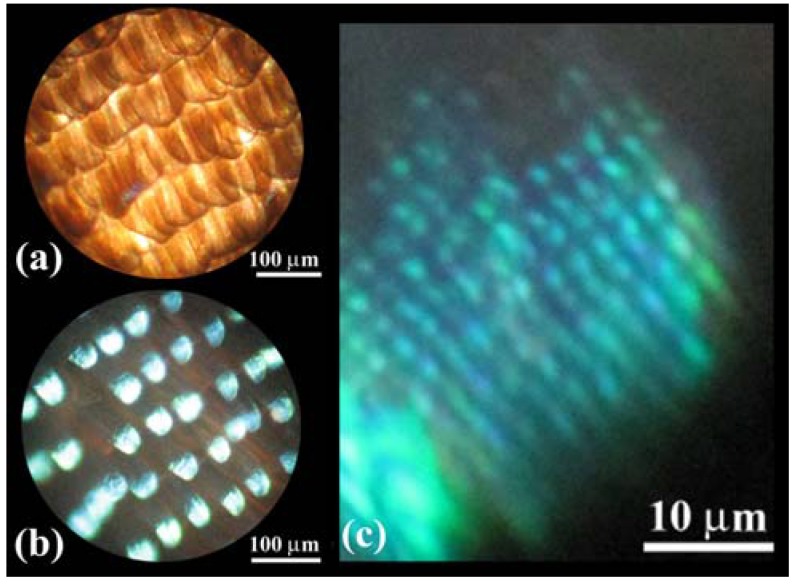
OM images of the dorsal wing scales of the male *C. ataxus* taken by white light. (**a**) Transmission image; (**b**) Reflection image; (**c**) Enlarged image of a scale in (b) (adapted from [14]).

Imafuku *et al*. [32] measured wing colors of *Chrysozephyrus* butterflies with a spectrophotometer, and reported that the dorsal wing surface of a male *C. ataxus* shows a strong reflectance when the specimen is tilted and that it appears green to the human eye, reflecting UV as well as green light. Table 2 was made using their results, especially the reflectance/wavelength curves displayed in Figure 7 in their paper. The green hues in Figure 1(c) correspond to the reflection of a very broad peak at 547 nm with a full-width at half-maximum (FWHM) of ~125 nm. In the measurement shown in Table 2, the incident light was applied to a wing piece, and the reflected light that returned in the same course was measured [32]. This means that the reflectance measured was from the surfaces whose normal is parallel to the incident light. If the groove plates cause multi-reflection between the layers with air gaps, the reflection is observed when the following interference condition is satisfied:

2(*n*_c_*t*_c_*+ n*_a_*t*_a_) = *mλ*_p_ (2)

We can take *n*_c_ = 1.55 for the layer substance as mentioned in Section 3.1 and *n*_a_ = 1. Assuming *m* = 1 for the observed reflection peak of *λ*_P_ = 547 nm, (1.55*t*_c_ *+ t*_a_) = 274 nm, and the corresponding wavelengths of the reflection for *m* = 2 and *m* = 3 must be 274 nm and 182 nm, respectively. However, the observed peaks did appear neither at 274 nm nor at 182 nm in the spectrum measured by Imafuku *et al.* [32]. This suggests that the multiple layers in the groove plates cannot be considered as the structure creating the multiple interference but they must be incoherent with each other. This is natural because their surfaces may not be completely parallel to each other. Then, we consider the interference for one layer, where the reflection takes place under the following condition:

2*n*_c_*t*_c_ = (*m* + 1/2)*λ*_P_ (3)

**Table 2:** Wavelength λ_P_, FWHM and reflectance *r*_P_ of the peaks on the reflectance spectra, which were measured for the iridescent wings of the male and female *C. ataxus* butterflies by Imafuku *et al*. [32].

Sex	λ_P_ (nm)	FWHM (nm)	*r*_P_ (%)	λ_P_ (nm)	FWHM (nm)	*r*_P_ (%)	λ_P_ (nm)	FWHM (nm)	*r*_P_ (%)
Male	257 *	35 *	25 *	341 ± 5	89 *	39 *	547 ± 4	125 *	36 *
Female	252 *	30 *	13 *	395	134 *	43 *			

The values with asterisk were estimated from the curves in Figure 7 of Reference [32].

**Figure 8:**
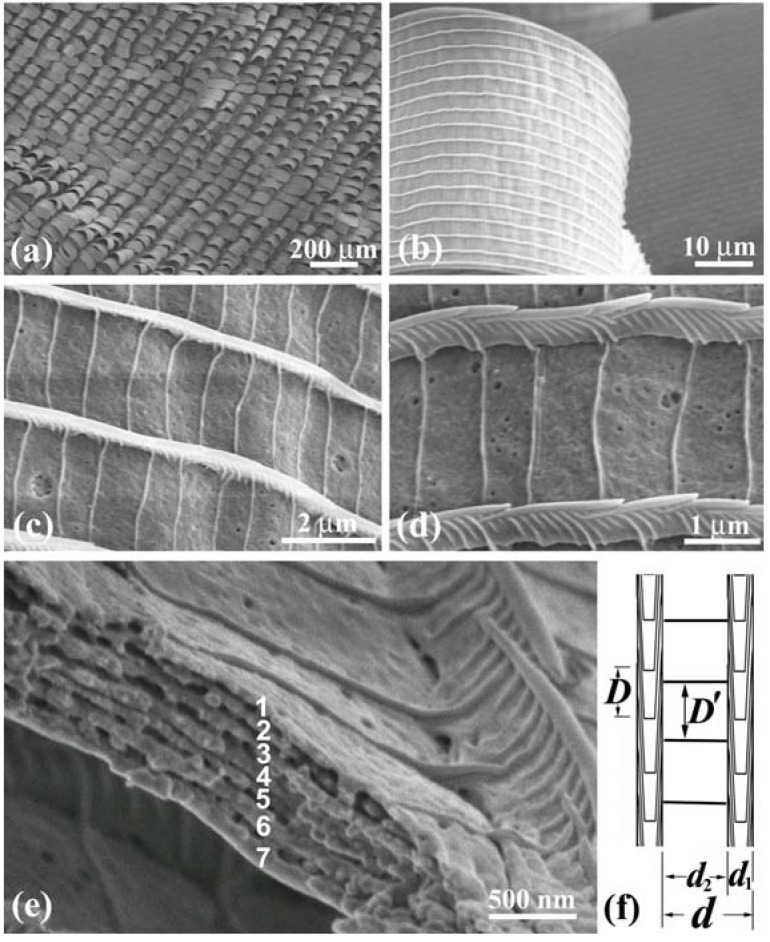
SEM images of the dorsal scales of the male *C. ataxus*. (**a**) Law-magnified image of curled scales; (**b**) A curled scale; (**c**) Ridges and cross ribs in the scale; (**d**) Enlarged image of the ridges and ribs; (**e**) Cross-section of a groove enclosed by the ridges and ribs, revealing seven piled layers; (**f**) Schematic of the fine structure of the scale (adapted from [14]).

Taking *m* = 1 for *λ*_P_ = 547 nm and *m* = 2 for *λ*_P_ = 341 nm, we obtain thicknesses of *t*_c_ = 265 and 275 nm, respectively. We thereby acquire a reasonable thickness of the flat groove layers of *t* = ~270 nm. Although we could not estimate the thickness from Figure 8(e) because the layer surfaces were not perpendicular to the imaging plane of SEM, it is not completely inconsistent with the images. The observed UV peak of *λ*_P_ = ~257 nm is then regarded as the reflection of *m* = 3. Thus, each layer reflects incoherently or kinematically with others, like a particle of the mosaic structure in X-ray diffraction, and the reflectance intensity is a simple sum of the reflection from each layer. Imafuku *et* *al*. [32] mentioned that the wing surface shows a strong reflectance when the specimen is tilted. This is simply explained as a geometric result of the increase of the area on curled scales that is normal to the incident light and of the number of the scales irradiated by the incident light as the specimen is tilted. When we observe the wing obliquely at a glancing angle of *θ*, the strong reflection is obtained if the diffraction condition

2*n*_c_·*t*_c_cos*θ*_c_ =(*m* + 1/2)*λ* (4)

is satisfied, where *θ*_a_ = 90° − *θ* and sin *θ*_a_/sin *θ*_c_ = *n*_c_. Since −1 ≤ sin *θ*_a_ = *n*_c_ sin *θ*_c_ ≤ 1 and 0 ≤ cos *θ*_c_ ≤ 1, taking *t* = ~270 nm the satisfied wavelength is calculated as ~558 ≥ λ ≥ ~426 nm for *m* = 1 and ~335 nm ≥λ ≥ ~256 nm for *m* = 2. The human eye perceives only the reflected light in a range of ~558 (green) > λ > ~426 nm (violet) in the glancing angle 180° ≥ *θ* ≥ 0. Each scale is highly curved as can be seen in Figure 8, so that some part of the scale satisfies the interference condition and gives a strong reflection, as seen in Figure 7(b). That is the color of the male *C. ataxus* butterfly in Figure 1(c).

There is a report on the curled scales in the wing of *Chrysiridia rhipheus* (the Madagascan sunset moth) [20]. From SEM observation of a thin width of *d*_1_ = 0.4 μm and a large separation of *d*_2_ = 3.5 μm of the ridge it is assumed that the reflection of the scale is mostly due to the basal layer (in grove) of the scale, and from cross-sectional transmission electron microscopy that the basal layer consists of air-cuticle alternate layers all over the scale. It concluded that this groove structure together with multilayer optical interference produces an unusual optical effect through an inter-scale reflection mechanism; thereby the wing color changes depending on light polarization. As a result, the coloration of the male *C. ataxus* dorsal wing is completely different from that of the *Chrysiridia rhipheus* although they have similar multiple layers on the basal flat areas between the ridges. It should be noted that the vivid coloration of *C. ataxus* is not ascribed to the multiple interference between the piled layers but to the interference between the top and bottom surfaces of each layer. The rays reflected from different layers are incoherent with each other.

**Figure 9:**
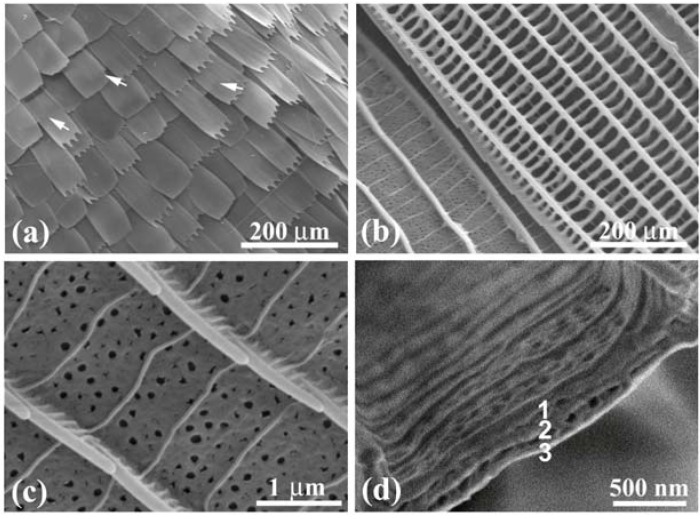
SEM images of the dorsal scales of the female *C. ataxus*. (**a**) Law-magnified image; (**b**) Ridges and cross ribs in a violet scale (left) and a dark brown scale (right); (**c**) Enlarged image of the ridges and ribs in the violet scale; (**d**) Cross-section of a groove plate enclosed by the ridges and ribs, revealing triple piled layers (adapted from [14]).

Figure 9(a) shows scales in a violet mark in the female’s dorsal forewing. A vein is observed near the upper right corner. Some scales in the cell are curled and the others are almost flat exhibiting the slit top tails. The image in Figure 9(b) comprises two scales: One has flat layers in the areas enclosed by the ridges and ribs (left) and the other has no layers (right). The former is a scale exhibiting violet hues, and the latter is a scale on the vein and similar to the scales exhibiting dark brown (Table 1). Figure 9(c) shows an enlarged image of the violet scale, whose structure is similar to that of the male’s scales in Figure 8 although it has many holes. As shown in Table 1, the ratio *d*_1_/*d* is small and *n* = 1 so that the contribution of the cuticles on the ridges to the color hues would be small. As seen in Figure 9(d), the groove plates take the multilayered structure of triple layers. We then estimated the thickness of the layers, similar to the calculation for the male wing, using the data shown in Table 2 and Equation (3). The observed strong intensity of *λ*_P_ = 395 nm with *r*_P_ = 43% can be regarded as the first order reflection (*m* = 1) from the layer with a thickness of *t*_c_ = ~191 nm. We do not take *t*_c_ = ~319 nm for *m* = 2 because no reflection corresponding to *m* = 1 appeared at 659 nm in the experiment [32]. The small thickness of the layers is confirmed by 1.5 kV SEM image in Figure 9(a), where the underlying scales are recorded through their upper scales as indicated by arrowheads. An FWHM as large as134 nm is attributed to a large variation of the thickness among layers and the curl of the scales. In any case the possible reflection in the overall angle 180° ≥ *θ* ≥ 0 occurs only for the rays in wavelength ranges of ~400 ≥ λ ≥ ~300 nm (*m* = 1) and ~240 ≥ λ ≥ ~180 nm (*m* = 2). Hence, the female wing’s marks look violet in the range of human visibility.

We thus gave a reasonable explanation for the coloration of the male and female *C. ataxus* butterfly’s wings on the basis of our SEM observation and optical data reported by Imafuku *et al.* [32].

### 3.3. Multilayered Microribs

**Figure 10:**
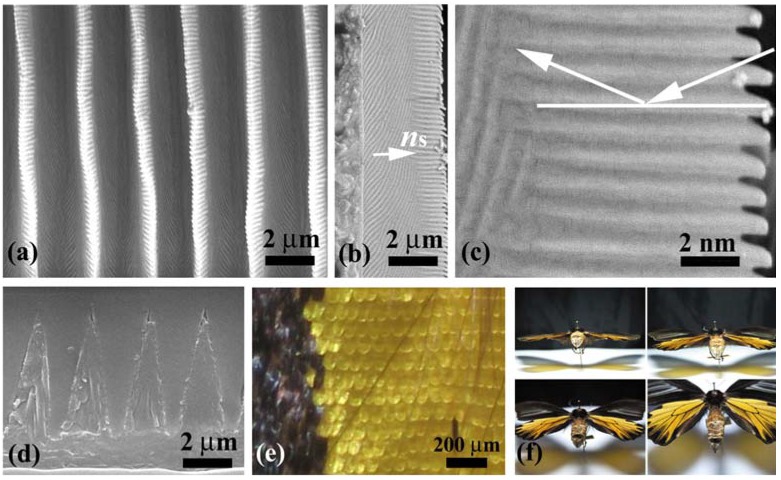
(**a**–**d**) SEM images of the yellow scales of the male *T. aeacus.* Top view of ridges (**a**); side-view of a ridge (**b**); enlarge image of the side view (**c**) and cross-section of four ridges (**d**); (**e**) Reflection OM images of the dorsal wing scales; (f) Photographs of the male *T. aeacus,* taken at different near-grazing incidences (adapted from [11]).

The yellow scales of *T. aeacus* macroscopically look similar in shape to the brown scales of *E. mulciber* and *S. charonda*. However, Figure 10(a–d) reveals that they are completely different in microstructure from these brown scales. The scale looks like a construction arranged with triangle bars, as seen in Figure 10(d) that shows a cross-sectional SEM image of the four ridges. The main difference is that there are no cuticles piled on the ridges (Figure 10(a)) but protrusions called “microribs” [4,10] are present on the sides of the triangular ridges (Figure 10(b,c)). Irregular gratings closer to the scale membrane are also seen. The microribs stand perpendicularly to the scale plane (as if ignoring their bent tops). The bases of the triangle bars are as long as *d*~1.8 μm and the spacing of the microribs D* is about 0.2 μm. Since there are no cuticles on the ridges, such an iridescence as observed in *E. mulciber* is not produced. It is known that *T. magellanus* (and also *T. Prattorum*), which closely resembles *T. aeacus,* however, exhibits a blue-green sheen when it is observed at near-grazing incidence [9]. The sheen is a result of the correlated diffraction and fluorescence in the backscattering iridescence that is caused by steeply-set multilayered microribs [10]. *T. aeacus* did not display a blue-green sheen such as demonstrated in Figure 1 in the paper by Vigneron *et al.* [10] when we viewed it from oblique angles (Figure 10(f)). In *T. magellanus*, the repeat period of the microribs D* is ~0.26 μm, and the slant angle of the microribs *θ*_B_* is ~54° with respect to the scale surface [9], or ~53°, which is exactly ~61° with respect to the ridge crest that is tilted by ~8° to the scale surface [10]. The slant of the multilayered microribs is the requirement for the backscattering iridescence. *T. aeacus* has the microrib layers perpendicular to the scale plane so that the backscattering diffraction hardly occurs from light with any incidence angle as indicated by the arrows in Figure 10(c). That is a reason why *T. aeacus* does not exhibit iridescence on the yellow scales unlike *T. magellanus.* As seen in Figure 1(e) and Figure 10(e) these scales are intrinsically yellow. This is confirmed by the yellow shadows of the wings, shown as transmitted images in Figure 10(f). The hues of the yellow color may be ascribed to the absorption and scattering of the incident rays by papiliochrome in the scales [9].

Black scales in the marginal area are also shown in Figure 10(e). The black scales are almost similar in structure to the dark brown **B4** scales at the edge of *E. mulciber’*s wing shown in Table 1. The difference in color is perhaps ascribed to a difference of the content of the pigment melanin.

## 4. Conclusions

Wing scales of several butterflies exhibiting selective wavelength iridescence from violet to green were investigated by SEM and optical reflectance measurement.

(1) The scales of the male *S. charonda* and *E. mulciber* butterflies have a highly tilted, multi-layered cuticle-air arrangement on the ridges, similar to *A. meliboeus.* This highly tilted, multi-layered cuticle-air arrangement, together with a grid that comprises the ridges aligned along the scale length and the cuticle layers spaced on the ridges, forms a three-dimensional blazed diffraction grating or a monoclinic lattice. The scales cause a limited-view iridescence, which is correlated with the human invisible zones due to UV reflection as well as a no reflection dark zone geometrically caused by tilting of cuticle layers. The multiple interference from the highly tilted, multi-layered cuticle-air arrangement illustrates the selective wavelength iridescence of the *E. mulciber*; where human eyes can detect the reflection in a range of about 510 (**green**) ~380 nm (**violet**).
(2) The iridescence from the *C**.** ataxus* originates from multilayers in the groove plates between the ridges and ribs. The vivid coloration is not ascribed to the multiple interference between the layers but to the interference between the top and bottom surfaces of each layer. The rays reflected from different layers are incoherent with each other. The human visible reflection from the male is limited to a wavelength range between ~560 (**green**) and 430 nm (**violet**) and that from the female is below ~400 nm (**violet**).
(3) Yellow scales in *T. aeacus*’s hindwing do not have any multilayered cuticle arrangement but have microrib multilayers on the sides of triangle ridges. The microrib layers are perpendicular to the scale plane so that they do not reflect any backscattering. That is a reason why the *T. aeacus* does not produce the iridescent sheen, which *T. magellanus*, belonging to the same genus, *Troides*, reflects with the slant microribs at near-grazing angle.

The coloration mechanisms of these scales are very simple so that they would provide us helpful hints to manipulate light in photoelectric devices, such as blue or UV LEDs and also to design photochemical crystal devices for internal conversion where the UV radiation is directly and effectively transformed into heat.
